# An Uncommon Cause of a Small-Bowel Obstruction

**DOI:** 10.1155/2017/1628215

**Published:** 2017-03-12

**Authors:** Ali Zakaria, Bayan Al Share, Issam Turk, Samira Ahsan, Waseem Farra

**Affiliations:** Department of Internal Medicine, Division of Pulmonology, Providence-Providence Park Hospital, Michigan State University College of Human Medicine, Southfield, MI, USA

## Abstract

Sarcoidosis is a systemic granulomatous disease of unknown etiology, characterized by the formation of noncaseating granulomas. Gastrointestinal (GI) system involvement that is clinically recognizable occurs in less than 0.9% of patients with sarcoidosis, with data revealing small intestine involvement in 0.03% of the cases. A high index of suspension is required in patients presenting with small-bowel obstruction and previous history of sarcoidosis. Establishing a definitive diagnosis of GI sarcoidosis depends on biopsy evidence of noncaseating granulomas, exclusion of other causes of granulomatous disease, and evidence of sarcoidosis in at least one other organ system. Treatment of GI sarcoidosis depends on symptomatology and disease activity. Herein, we are presenting a case of 67-year-old female patient who had acute small-bowel obstruction at the level of jejunum with postoperative histopathologic evidence of noncaseating granulomatous inflammation with multinucleated giant cells, consistent with sarcoidosis.

## 1. Introduction

Sarcoidosis is a systemic granulomatous disease of unknown etiology, characterized by the formation of noncaseating granulomas. Gastrointestinal (GI) involvement is very rare and can occur in a patient with known sarcoidosis or as the initial manifestation of the disease. Small-bowel obstruction secondary to sarcoidosis has been described in few case reports. Herein, we are reporting a case of 67-year-old female patient who had acute small-bowel obstruction at the level of jejunum with postoperative histopathologic evidence of noncaseating granulomatous inflammation consistent with sarcoidosis.

## 2. Case Report

**Figure 1:**
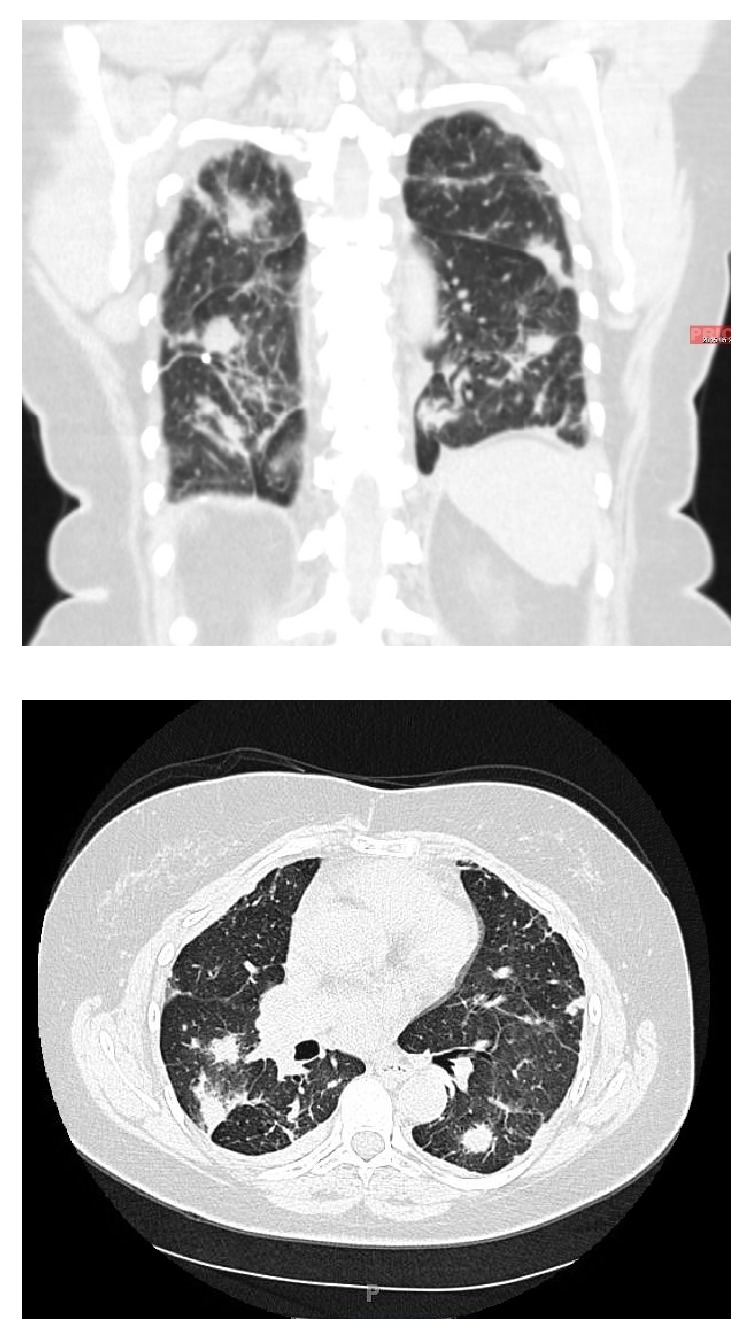
Computed tomography (CT) scan of the chest reveals diffuse parenchymal nodularity, with no honeycombing, hilar, or mediastinal adenopathy, consistent with stage III sarcoidosis.

**Figure 2:**
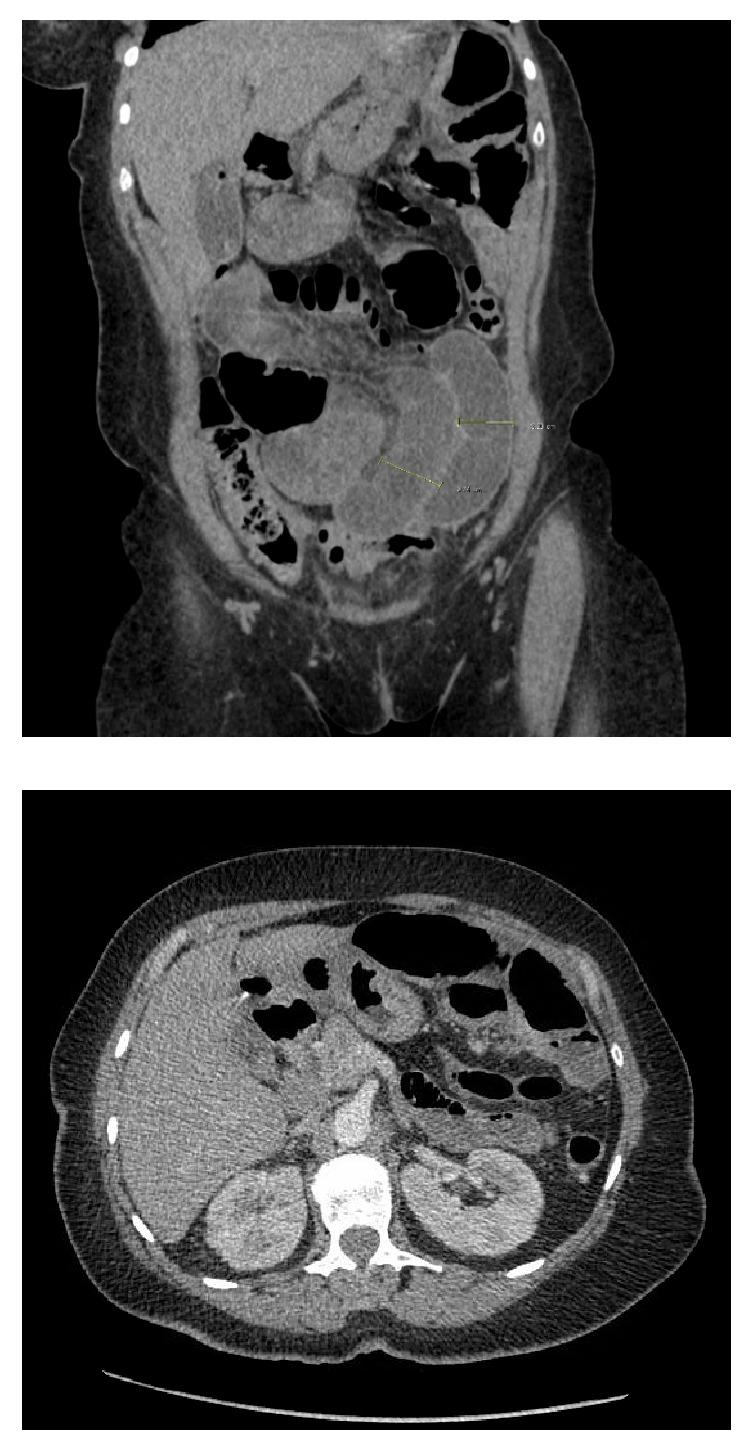
Computed tomography (CT) scan of the abdomen demonstrated early complete bowel obstruction at the level of midjejunum.

A 67-year-old female with previous medical history of inactive pulmonary sarcoidosis (Figure 1), hypertension, and impaired glucose tolerance, presented to emergency department with five-day history of nausea, vomiting, abdominal pain, and constipation. On physical examination she was alert, oriented, and not in acute distress. Her vital signs were as follows: Temp 36.7°C; pulse 93 bpm; respiratory rate 18 bpm; blood pressure 147/85 mmHg; and O_2_% of 98% on room air. Her abdomen was distended and mildly tender over the epigastric area, with no evidence of organomegaly. Her bowel sounds were hyperactive. Initial imagining studies with abdominal X-ray revealed dilated small-bowel loops with multiple scattered air fluid levels suggesting partial versus early complete small-bowel obstruction. A contrasted computed tomography (CT) scan of the abdomen and pelvis demonstrated multiple dilated loops of proximal small bowel with no definitive transition point and no intraperitoneal free air or fluid identified, a picture suggestive of a high-grade small-bowel obstruction (Figure 2).

**Figure 3:**
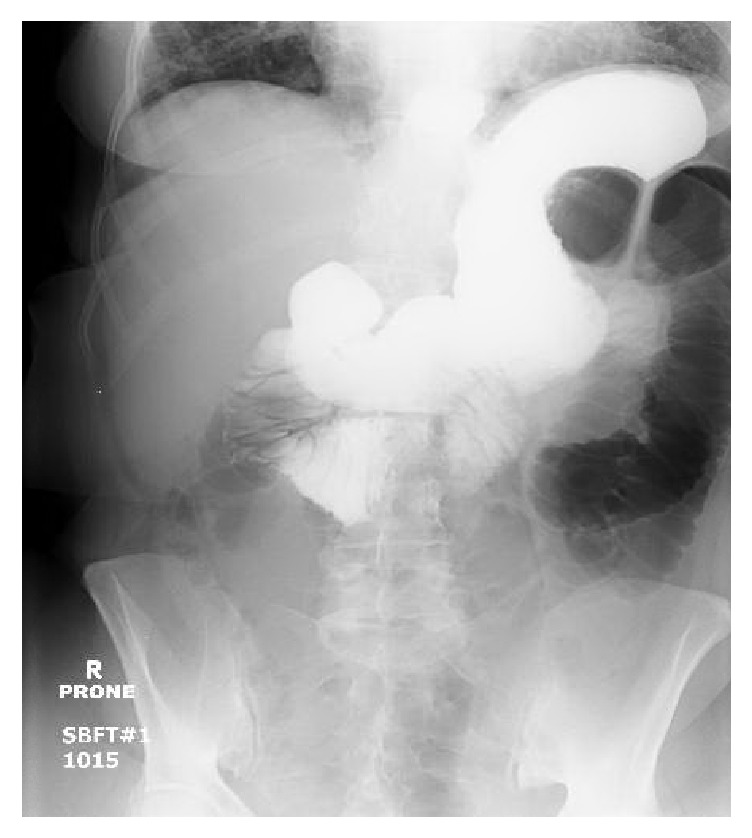
Gastrografin small-bowel follow-through demonstrated failure of contrast to pass into the colon.

She was managed conservatively with bowel rest, nasogastric tube, and IV fluids. Her symptoms failed to improve, so gastrografin small-bowel follow-through was performed and demonstrated failure of contrast to pass into the colon (Figure 3).

**Figure 4:**
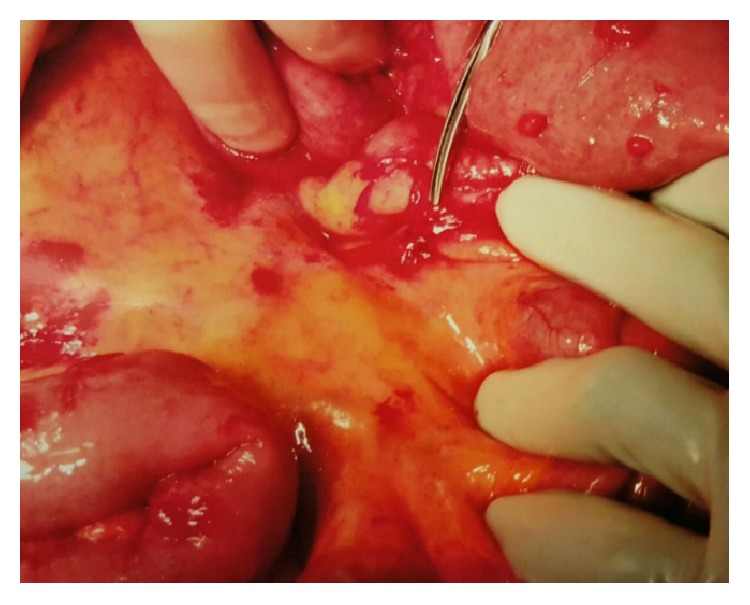
Intraoperative demonstration of the skin tag appearing lesion at the level of midjejunum causing inflammatory adhesions.

**Figure 5:**
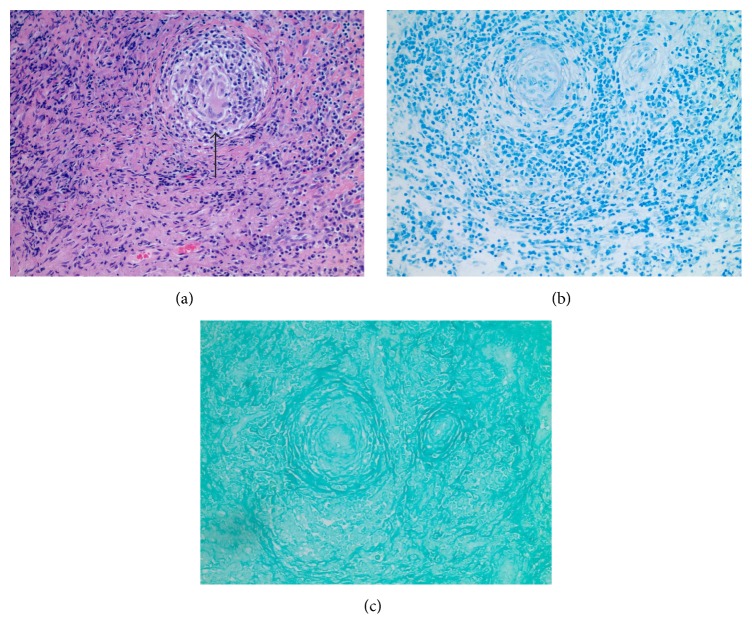
Histopathology of the resected small-bowel lesion. (a) H&E stain revealed lymphohistiocytic noncaseating granuloma with multinucleated giant cells (black arrow). (b) Negative for acid-fast bacilli (AFB) stain. (c) GMS stain negative for fungal infection.

General surgery was consulted and diagnostic laparoscopy was performed and revealed multiple dilated bowel loops which revealed diffuse skin tag appearing lesions on the antimesenteric surface of the midjejunum causing inflammatory adhesions to the mesentery (Figure 4). The bowel was completely viable with no additional lesions in the liver, stomach, or visible parts of the colon. Histopathologic examination of tissue biopsy revealed lymphohistiocytic noncaseating granulomatous inflammation with multinucleated giant cells, consistent with sarcoidosis (Figure 5). Further evaluation revealed negative acid-fast bacilli and fungal and viral tissue cultures. Her postoperative course was uncomplicated and she was discharged on prednisone taper dose with close outpatient follow-up.

## 3. Discussion

Sarcoidosis is a systemic granulomatous disease of unknown etiology, characterized by the formation of noncaseating granulomas. Gastrointestinal (GI) system involvement that is clinically recognizable occurs in 0.1–0.9% of patients with sarcoidosis, with one study revealing postmortem small intestine involvement on autopsy in 0.03% of the cases [1, 2]. It usually occurs in patients in their fifth or sixth decade of life with evidence of multisystem sarcoidosis in approximately one-half of patients. The most common manifestation is nonbloody diarrhea with colicky abdominal pain. It can also present with nonspecific constitutional symptoms, obstruction, and/or intestinal hemorrhage, a presentation that can mimic other diseases such as Crohn's disease. Other rare manifestations include mesenteric venous insufficiency due to pressure from enlarged sarcoid lymphadenopathy, megaloblastic anemia caused by terminal ilium infiltration, and protein-losing enteropathy [3–6]. Establishing a definitive diagnosis of GI sarcoidosis depends on three components: (1) biopsy evidence of noncaseating granulomas in the symptomatic or incident organ, (2) exclusion of other causes of granulomatous disease, particularly mycobacterial, fungal, and parasitic infections, and (3) clinical, radiographic, and optimally histopathologic evidence of sarcoidosis in at least one other organ system [7]. Imaging studies using computed tomography scan considered appropriate initial step in diagnosing patients who present with abdominal pain, while, in those who present with prominent diarrhea, endoscopy is more appropriate. Treatment of GI sarcoidosis depends on symptomatology and disease activity; asymptomatic patients can be monitored without active therapy, while symptomatic patients with substantial organ involvement and granulomatous inflammation on tissue biopsy should be treated with corticosteroid for six to eight weeks or until a response to therapy is noted and then gradually tapered, over a period of approximately six months [7]. The optimal duration of corticosteroid treatment for gastrointestinal sarcoidosis is not known, but experts recommend treating the initial manifestation for at least one year. Other treatments such as surgical intervention may be necessary in patients with bowel obstruction, perforation, or massive hemorrhage. Monitoring of GI sarcoidosis is achieved clinically and radiographically, but there is no evidence for the role of serum angiotensin converting enzyme or serum interlukin-2 receptor in monitoring [8, 9].

## 4. Conclusion

Gastrointestinal (GI) sarcoidosis is very rare and can occur in a patient with known diagnosis or as initial presentation. A high index of suspension is required in patients presenting with small-bowel obstruction and previous history of sarcoidosis.
